# Prevalence of allergic conjunctivitis among basic school children in the Kumasi Metropolis (Ghana): a community-based cross-sectional study

**DOI:** 10.1186/s12886-015-0053-8

**Published:** 2015-07-03

**Authors:** David Ben Kumah, Seth Yaw Lartey, Felix Yemanyi, Evans Gyimah Boateng, Emmanuel Awuah

**Affiliations:** Department of Optometry and Visual Science, Kwame Nkrumah University of Science and Technology, Kumasi, Ghana; Department of Eye, Ear, Nose and Throat, Komfo Anokye Teaching Hospital, Kumasi, Ghana

**Keywords:** Allergic conjunctivitis, Prevalence, Community-based

## Abstract

**Background:**

There seems a preponderance of hospital-based studies on the prevalence of Allergic Conjunctivitis (AC) compared to community-based ones, particularly among children in Ghana and Africa as a whole. Meanwhile, literature supports the possibility of underdiagnosing AC in the hospital setting; exponentially so when males generally have poor hospital-attending behavior. This may lead to underestimation of the true burden of AC. Consequently, the purpose of the current community-based study was to determine the prevalence of AC among basic school children in the Kumasi Metropolis, while identifying its associated symptoms.

**Methods:**

A cross-sectional community-based study involving 1571 students from 11 basic schools (Primary and JHS) participated in the study. Data collection started in November 2011 and was completed in March 2014. After history taking, subjects underwent a battery of tests; visual acuity, objective refraction, anterior and posterior segments examination with a slit-lamp and a direct ophthalmoscope respectively.

**Results:**

The prevalence of AC was 39.9 %. The mean (±SD) age of participants was 8 ± 0.65 years. AC was significantly associated with gender (p < 0.05), but not with age (p > 0.05). A total of 70 % of the students with AC never had any form of treatment.

**Conclusions:**

AC is an endemic ocular disease among basic schools in the Kumasi metropolis and therefore calls for pragmatic and proactive measures to reduce its burden and effects on its victims. Public health measures may be required to help reduce the burden associated with this condition.

## Background

Allergic conjunctivitis (AC) is an inflammation of the conjunctiva due to allergy [1–3]. AC ranges from more prevalent non-sight threatening conditions like seasonal allergic conjunctivitis (SAC), perennial allergic conjunctivitis (PAC) and giant papillary conjunctivitis (GPC) to less prevalent sight-threatening ones such as vernal keratoconjunctivitis (VKC) and atopic keratoconjunctivitis (AKC) [4]. Generally, it is a type 1 immunoglobulin E (IgE) mediated hypersensitivity reaction while cell-mediated T-helper cells-2 (Th-2) are implicated in some types [4, 5]. AC involves a cascade of events which begins when mast cells become unstable and subsequently break down [6]. How does this degranulation of mast cells occur? The conjunctiva mounts an antigen-specific response with Th-2 upon contact with an allergen, releasing cytokines and producing IgE. IgE then binds to mast cells which break down; releasing histamines, prostaglandins, platelet-activating factor, more cytokines and other intermediaries. The signs and symptoms of AC result from the activation of inflammatory cells by these intermediaries [7]. For instance, when histamine binds to H1 receptors on nerve endings, it causes the symptom of itchiness; when it becomes attached to H2 receptors found on blood vessels in the conjunctiva, it produces vasodilation and lacrimation. The process could be heightened and become chronic in nature with progressive recruitment of neutrophils and eosinophils by mast-cell derived cytokines and Th-2 cytokines respectively [8, 9]. This is what leads to a probable development of papillae in some patients [5]. It is difficult finding a single cause for AC because it is believed to result from an interplay of varying causative factors like genetics, air pollution in urban areas, pets, warmer climates and early childhood exposure [10, 11]. For instance, in Ghana, Obeng et al. [12] found out that some food substances like groundnut (peanut) and pineapple could trigger AC. Ocular discomfort associated with AC include itching, redness, tearing, pains, burning sensation, lids and conjunctival edema together with foreign body sensation. These symptoms would almost invariably affect academic performance and the vision-oriented quality of life of victims resulting in morbidity and loss of productivity [13–17].

While older population studies estimate AC prevalence of 15–20 % globally, more recent studies report rates as high as 40 % [18]. This probably supports a continuing increase in prevalence pattern of AC [13] despite treatment options like lubricating agents, vasoconstrictors, antihistamines, mast cell stabilizers and topical steroids [19]. Consequently, there has been a shift to immunotherapy especially in the United States as an alternative treatment modality [13]. Additionally, as a remedy, O’Brien et al. [19] recommends a multifaceted management regimen consisting of patient education and lifestyle modification.

However, because AC is usually underdiagnosed and subsequently undertreated, a related prevalence studies would probably provide a true picture of the burden, while enhancing informed decision on pragmatic management modalities. Moreover, this would possibly assure effective and efficient health resource allocation in a bid to possibly improve upon the quality of life of the affected [18].

While there is a preponderance of hospital-based prevalence studies of AC in Ghana [20, 21] and Africa [22, 23], the same could possibly not be said of community-based studies. Accordingly, this study, virtually the first Ghanaian community-based studies, had the purpose of determining the prevalence of AC among basic school children in the Kumasi Metropolis together with identifying its main presenting symptoms.

## Methods

### Recruitment of study subjects

A multistage stratified clustered random sampling was used in selecting a sample of 1571 students from 11 basic schools (Primary and Junior High School [JHS]) in the Kumasi Metropolis from November to March in 2012, 2013 and 2014 respectively. Study was approved by the Ethics Review Board of the Department of Optometry and Visual Science, Kwame Nkrumah University of Science and Technology, and carried out in accordance with the declaration of Helsinki. After approval from the Metropolitan Educational Directorate and the school heads, parental consent in both written and verbal forms was sought for their wards to be included in the study. The students were informed about study’s objectives and they could withdraw thereof if they wished so to do.

### Data collection procedures

A structured questionnaire was used to collect history on subjects’ demographics and medical data in the respective schools. Distance and near visual acuities were measured with Snellen literate chart. Objective refraction was performed for subjects with VA ≤ 6/12. This was followed with a comprehensive anterior and posterior segment eye examination by an ophthalmologist (Seth Yaw Lartey^2^) with slit-lamp biomicroscope (SL 500 Shin Nippon, Ajinomoto Trading Inc., Tokyo, Japan) and a direct ophthalmoscope respectively.

Allergic conjunctivitis was diagnosed by symptoms of bilateral itchiness and either burning sensation, tearing, ropy/clear mucinous discharge, or photophobia. The ocular signs hinged on the presence of at least two of these: papillae, redness, brownish limbal hyperpigmentation, visible limbal tranta spots and chemosis [22, 24].

### Data analysis

Epi Info software version 3 (Centre for Disease Control, Atlanta, Georgia, US) was used both in calculating study’s sample size and analyzing collected data, using descriptive and distributional statistics. To determine significant associations in the categorical variables (allergic conjunctivitis, gender, and age-group), Chi-Square (×2) test was used. The 5 % significance level was used in all analysis.

## Results

Of a total population of 5950 students, 1571 representing 26.4 % were recruited; 838 (53.3 %) were males while 733 (46.7 %) were females with female/male ratio being 1:1.14. Their ages ranged from 5 to 16 years (mean ± SD, 8 ± 0.65 years).

The prevalence of Allergic Conjunctivitis (AC) in the schools randomly sampled is shown in Table 1. Of a total of 1571 students, 626, representing 39.9 % had AC. Table 2 illustrates the prevalence of AC by age and gender. Out of the 626 diagnosed cases of AC, 56.7 % (355) were females while 43.3 % (271) were males. Students aged 13–16 years recorded the highest number of cases (250), representing 39.9 %. In Table 3, the distribution of the major symptoms and signs of AC is illustrated; students suffered multiple symptoms.

**Table 1:** Prevalence of Allergic Conjunctivitis in the schools randomly sampled

SN	Name Of School	Number screened	Cases of Allergic Conjunctivitis (%)
1.	Ayeduase M.A. Primary and JHS (AY)	116	101 (87.0 %)
2.	Prempeh College Basic School (PC)	198	128 (64.6 %)
3.	Amankwatia M.A. Basic school A (AM)	231	57 (24.7 %)
4.	State Boys school (SB)	141	72 (51.1 %)
5.	Wesley College Practice School	114	90 (44.8 %)
6.	Asem Mixed (AS)	75	13 (17.3 %)
7.	State Experimental School (SE)	285	57 (20.0 %0
8.	Old Tafo M.A. Primary and JHS (OT)	122	27 (22.1 %)
9.	Aprade M.A. Primary and JHS (AP)	14	2 (14.3 %)
10.	St. Theresa R.C. Primary and JHS (TH)	160	69 (43.1 %)
11.	St. Paul Anglican Primary and JHS (PA)	115	10 (8.7 %)
Total		1571	626 (39.9 %)

**Table 2:** Prevalence of Allergic Conjunctivitis by age and gender

Age Group (Years)	Frequency		
	Males	Females	Total
5–8	83	108	191 (30.5 %)
9–12	81	104	185 (29.6 %)
13–16	107	143	250 (39.9 %)
Total	271 (43.3 %)	355 (56.7 %)	626 (100 %)

**Table 3:** Distribution of major symptoms and signs of Allergic Conjunctivitis

Symptoms	Frequency (%)
Itching	100 %
Redness	71.2 %
Ropy/Stringy Mucinous Discharge	66.3 %
Grittiness	60.5 %
Tearing	39.3 %
Photophobia	29.7 %
Clear Mucinous Discharge	17.9 %

Meanwhile, 68.3 % (428) of the diagnosed cases of AC were mild, while 31.7 % (198) were severe. Moreover, while 70.0 % (438) of them never had any form of treatment for AC, 17.0 % (106) had had treatment in the past; only 13.0 % (82) were receiving treatment (Table 4).

**Table 4:** Distribution of treatment status for Allergic Conjunctivitis

Treatment status	Frequency (%)
Currently receiving treatment (≤1 month)	82 (13 %)
Received treatment in the past (>1 month)	106 (17 %)
Never had any form of treatment	438 (70 %)

## Discussion

There are more Ghanaian studies on the prevalence of Allergic Conjunctivitis (AC) in the hospital setting than community-based ones [20, 21]. However, the former may not give a true picture of the burden of AC due to probable under-diagnosis [18]. Therefore, the current community-based cross-sectional study had the purpose of determining the prevalence of AC among basic school children in the Kumasi Metropolis, Ghana. AC was diagnosed by symptoms of bilateral itchiness and either burning sensation, tearing, ropy/clear mucinous discharge, or photophobia. The concurrent ocular signs were on the presence of at least two of these: papillae, redness, brownish limbal hyperpigmentation, visible limbal tranta spots and chemosis [22, 24].

The prevalence of AC was found to be 39.9 %, representing 626 out of 1571 students (Table 1) supporting a general continuing increase in its burden [13]. However, this is somewhat higher when compared to previous hospital-based studies [20, 22, 23, 25] except Adenuga et al. [26] who found the prevalence of AC to be 42 %. For example, Malu et al. [22] had 32 % as the prevalence of AC among patients who presented to an eye hospital in Nigeria. It is possible the lower prevalence of AC in hospital-based studies might be due to under-diagnosis of the condition [18]. Moreover, the mean (±SD) age of participants in Abokyi et al. [20], with AC prevalence 9.1 %, was 21.92 ± 18.29 years compared to 8 ± 0.65 years in the current study. Since AC is generally known to be a disease of children and young adults [20, 27], the lower mean age of the students in this study might contribute to the comparatively higher prevalence.

Again, Hamilton et al. [28] noted from 26 health practitioners that males were worse than females in attending hospitals for probable diagnosis and subsequent treatment of ailments. Consequently, hospital-based studies on prevalence of AC could most likely lead to an underestimation and possibly give credence to the higher AC prevalence in the current study.

Meanwhile, in their community-based studies among school children, Kumah et al. [29] and Abah et al. [30] found the prevalence of AC to be 12.1 % and 7.3 % respectively. Though the authors did not state the timing of their respective studies, the higher prevalence of AC in the current study is probably because of its timing in the dry season (November to March) where there is usually dust and pollen in the air [23]. Epidemiologic studies have confirmed exposures to particulate matter (air pollutants) as contributing to AC, more so when the conjunctiva is in direct contact with the atmosphere. For instance, upon exposing the conjunctiva of their study subjects to diesel exhaust, Fujishima et al. [31] found a significant upregulation of inflammatory cells like interleukin-6 and intercellular adhesion molecule-1 responsible for AC. Most of these air pollutants like urban dust contain polycyclic aromatic hydrocarbons which have been implicated in at least allergic lung diseases [32]; might not be different for AC.

Again, differences in study designs and sample sizes could render an explanation to the observed variations in AC prevalence. Moreover, it could be due to the recent general progressive increase in the prevalence pattern of AC [13] probably suggesting a global increase in air pollution.

As shown in Table 1, schools with non-dusty compounds like St Paul’s Anglican, Aprade M.A. and Asem mixed with the exception of State Experimental had lower respective prevalence of AC than those with dusty compounds. This is because of the probable association between AC and dust or sand [20, 23, 33].

The population of subjects diagnosed with AC consisted of 271 (43.3 %) males and 355 (56.7 %) females (Table 2). This is in consonance with several studies [20, 22, 34] with the exception of Marback et al. [35] who found the otherwise. Despite the fact that there were more males (53.3 %) than females (46.7 %) in the study sample (Table 1), females were more likely to be predisposed to AC than males (p < 0.05). Though the question of which gender is more predisposed to AC is a controversial one [36], the differences in the genetic composition of both sexes could probably be responsible for the observed difference [20].

Besides itchiness being reported by all respondents with AC, redness (71.2 %), ropy discharge (66.3 %) and grittiness (60.5 %) were the next commonest symptoms/signs (Table 3). Itching is a frequent and almost invariably indicates that the inflammation of the conjunctiva is allergic in origin [20]. Though dry eye may present with almost all the symptoms of AC; opposed to AC, its symptoms are usually associated with visual task [37] and characteristic staining [38]. The itching provokes eye-rubbing which may be responsible for some of the subsequent symptoms [2]. The discomfort, photophobia and itching may divert a child’s attention from the teacher to rubbing the eye to relieve the symptoms and this may affect the process of learning. It was noted that 70.0 % of the students diagnosed never had any form of treatment. This might be due to the fact that guardians possibly did not consider their children’s itchy eyes as needing treatment. Perhaps, the respective guardians saw the symptoms of AC as normal; poverty could also be a probable factor.

## Conclusion

Allergic Conjunctivitis is an endemic ocular disease amongst children of school going age in the metropolis and therefore calls for pragmatic and proactive measures to reduce its effects on these students.
